# Production of N^α^-acetyl Tα1-HSA through *in vitro* acetylation by RimJ

**DOI:** 10.18632/oncotarget.20259

**Published:** 2017-08-14

**Authors:** Jing Chen, Haibin Li, Tao Wang, Shuyang Sun, Jia Liu, Jianhua Chen

**Affiliations:** ^1^ School of Life Science and Technology, China Pharmaceutical University, Nanjing 210009, China; ^2^ Department of Neurosurgery, Shanghai 5th People's Hospital, Shanghai Medical College, Fudan University, Shanghai 200240, China; ^3^ Overseas Education College, Nanjing Tech University, Nanjing 211816, China

**Keywords:** thymosin alpha 1, human serum albumin, RimJ, acetylation, bioactivity

## Abstract

Thymosin alpha 1 (Tα1) is an important immunomodulating agent with various clinical applications. The natural form of Tα1 is *N^α^*-acetylated, which was supposed to be related to *in vivo* stability of the hormone. In this study, fusion protein Tα1-HSA was constructed and expressed in *Pichia pastoris*. RimJ, a *N^α^*-acetyltransferase from *E.coli*, was also overexpressed and purified to homogeneity. *In vitro* acetylation of Tα1-HSA in the presence of RimJ and acetyl coenzyme A resulted in *N^α^*-acetyl Tα1-HSA. The *N^α^*-acetylation was determined by LC-MS/MS. Kinetic assay indicated that RimJ had a higher affinity to desacetyl Tα1 than to Tα1-HSA. Bioactivity assay revealed fully retained activity of Tα1 when the hormone was connected to the N-terminus of the fusion protein, while the activity was compromised in our previously constructed HSA-Tα1. With fully retained activity and N-terminal acetylation, *N^α^*-acetyl Tα1-HSA was expected to be a more promising pharmaceutical agent than Tα1.

## INTRODUCTION

Thymosin alpha 1 (Tα1), an acidic thymic peptide consisting of 28 amino acids, was first described and characterized by Goldstein et al. [1]. As an endogenous regulator of both innate and adaptive immune systems, Tα1 has been shown to trigger maturational events in lymphocytes, to augment T-cell function, and to promote reconstitution of immune defects [2, 3]. Extensive clinical studies have been conducted to support the role of Tα1 in various indications. The versatility of Tα1 has aroused great interest in pharmaceutical industry. ZADAXIN, developed by SciClone Pharmaceutcials, has been marketed in more than forty countries as the first immune-boosting synthetic peptide for a variety of indications, including Hepatitis B and liver cancer.

One of the biggest challenges for biotechnical production of Tα1 by genetic engineering is degradation of the small peptide by proteases in the host cell, leading to decreased production and interference in isolation from degraded fragments [4]. Most of the strategies aiming at circumventing the above challenge follow the same pattern of production of a larger precursor and subsequent cleavage to release Tα1. Esipov *et al.* reported fused expression of Tα1 and thioredoxin followed by proteolytic cleavage of the precursor [5]. Expression of concatemer Tα1 gene of 6 repeats facilitated purification by increasing molecular size, and Tα1 monomer was released after hydroxylamine incision [6]. Fusion protein of Tα1-Intein was also successfully constructed to release Tα1 after intein-mediated N-terminal cleavage [7, 8]. All these approaches of cutting a bigger precursor into Tα1 suffer from low cleavage efficiency, imprecise or non-specific incision and expensive cost of the cutting enzymes. In addition, the harsh conditions required for cutting by chemical reagents such as hydroxylamine may structurally modify the target peptides.

In contrast, fusion of Tα1 with a partner that doesn't require subsequent cleavage is a more promising strategy. Human serum albumin (HSA) is the most abundant plasma protein (35–50 g/L human serum). When fused with target protein, HSA usually confers prolonged half-life [9], improved efficacy [10], and reduced toxicity [11]. In our previous work, two fusion proteins, HSA-Tα1 and HSA-linker-Tα1, were constructed and expressed in recombinant *Pichia pastoris* [12]. Both fusion proteins showed comparable bioactivity with Tα1 and improved pharmacokinetic profiles with prolonged half-life. Since HSA is connected to the N-terminus of Tα1, Tα1 in the two fusion proteins were desacetylated, while natural Tα1 and the commercialized thymosin α1 Zadaxin are *N*^α^-acetylated. Although desacetyl thymosin α1 is known to show biological activity equivalent to that of the native hormone, it is less stable *in vivo* [13]. There are several methods, chemical or biochemical, available for the production of *N^α^*-acetylated Tα1. Esipov *et al.* reported *in vitro N*^a^-acetylation of Tα1 by acetic anhydride [5]. Co-expression of the target protein with *N*^α^-acetyltransferase from *Escherichia coli* such as RimJ [14] and RimL [15] as well as *N^α^*-acetyltransferase from *Sulfolobus solfataricus*[16] also resulted in partial acetylation of Tα1.

In this study, a fusion protein Tα1-HSA with a native N-terminus of Tα1 was constructed and expressed in *P.pastoris*. N-terminal acetyl transferase RimJ was overexpressed in *E.coli* and purified to catalyze N-terminal acetylation of Tα1-HSA in the presence of acetyl coenzyme A. With fully retained activity and N-terminal acetylation, *N^α^*-acetyl Tα1-HSA was expected to be a more promising pharmaceutical agent than Tα1 and the previously obtained fusion proteins.

## RESULTS AND DISCUSSION

### Construction of expression vector pPICZαA/Tα1-HSA

In the fusion protein, HSA was connected to the C-terminus of Tα1 such that the N-terminus of Tα1 was available for acetylation. Tα1-HSA gene was introduced downstream of the gene coding for a-factor secretion signal peptide in pPICZαA. In order to express protein with a native N-terminus, Tα1-HSA gene was cloned flush with a Kex2 cleavage site (Supplementary Figure 1). After double digestion and insertion into pPICZαA, positive clones of *E.coli* Top 10 were selected by using 100μg/ml Zeocin and confirmed by DNA sequencing. The constructed expression vector pPICZαA/Tα1-HSA was amplified, extracted and then linearized with *Sac* I. The linear plasmid DNA was transformed into competent *P.pastoris* GS115 prepared by treatment with lithium chloride. After cultivation on YPD agar plate supplemented with zeocin at 30°C for 48-72 h, positive transformants were selected and confirmed by DNA sequencing.

### Production and purification of Tα1-HSA in *P. pastoris* GS115

**Figure 1:**
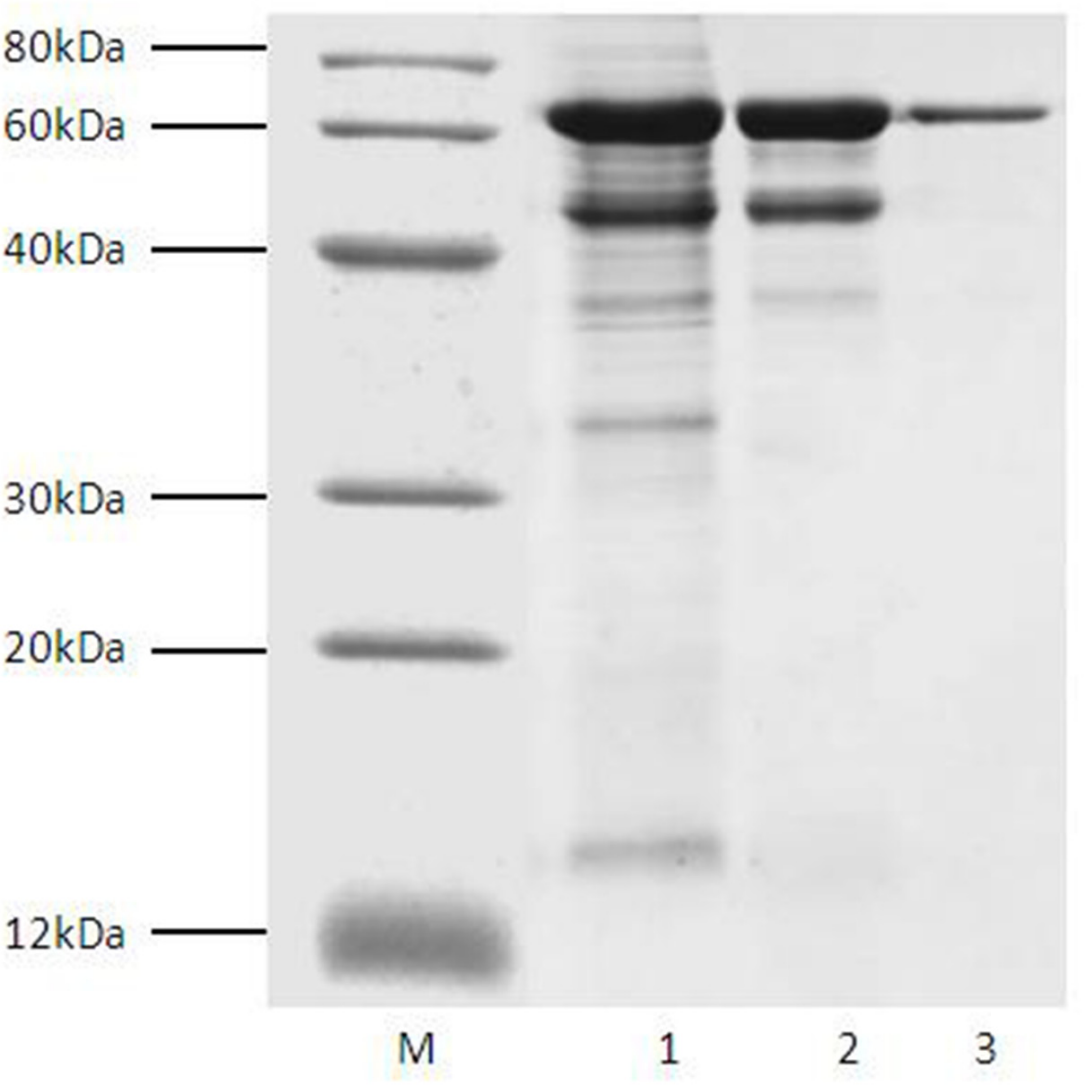
SDS-PAGE analysis of fractions during Tα1-HSA purification Lane M, marker; Lane **(1)** broth supernatant after ultrafiltration with Millipore Cogent M1 Tangential Flow Filtration System (molecular weight cutoff, 30kDa). Lane **(2)** fractions eluted from Capto™ MMC column; Lane **(3)** fractions eluted from Blue-Sepharose™ 6 Fast Flow column.

In order to increase the recombinant expression of Tα1-HSA, induction time (1-7 days) and methanol concentration (0.5-3.0%) were optimized in shake-flask cultures. As shown in Supplementary Figure 2 and Supplementary Figure 3, the optimum induction time was 6 to 7 days, and induction with 1.5% methanol was the most favorable. Because of secretory production led by α-factor, Tα1-HSA could be purified from the supernatant of fermentation broth. Compared with intracellular production, secretory production is more favorable to downstream purification because of less interfering proteins existing in the supernatant. In addition, native N-terminus could be obtained after cleavage of the signal peptide. After a series of purification steps including ultrafiltration, weak cation exchange and affinity chromatography with Blue-Sepharose Fast Flow, the purified protein gave a single band on SDS-PAGE stained with Coomassie brilliant blue R-250 (Figure 1). Its molecular mass was estimated to be around 70 kDa, which was very close to the theoretical molecular weight of the fusion protein.

### Construction, production and purification of RimJ

Acetylation of proteins is catalyzed by a variety of acetyltransferases that transfer acetyl groups from acetyl-coenzyme A to either the α-amino group of the N-terminal residues orε-amino group of lysine residues at various positions [17]. For the *in vitro* acetylation of Tα1-HSA, expression vector pET-28a(+)/RimJ was constructed and transformed into competent host cell *E.coli* BL21. Positive transformants were screened by 25μg/ml kanamycin and the target gene was confirmed by double digestion and DNA sequencing.

**Figure 2:**
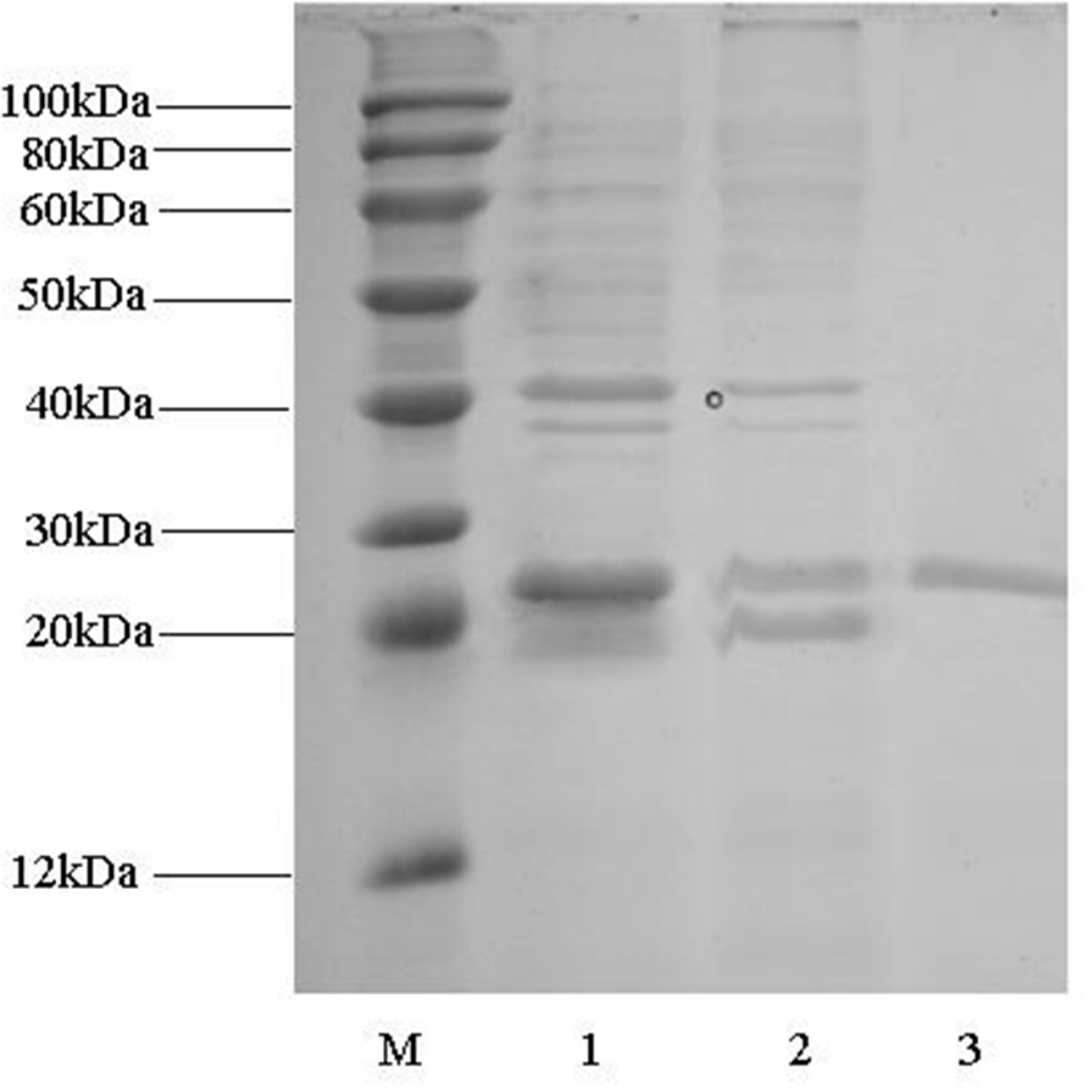
SDS-PAGE analysis of fractions during Rim J purification Lane M: marker; lane **(1):** supernatant after sonication; lane **(2):** fractions eluted from Capto™ MMC column; lane **(3):** fractions eluted from Ni^2+^ chelating column.

After cultivation at 37°C overnight, cell pellet was obtained after centrifugation and crushed by sonication. A substantial amount of RimJ was overexpressed in *E. coli* BL21 in the soluble form although inclusion body of RimJ was also detected. Since a N-terminal hexahistidine tag was fused with RimJ, a two-step purification containing cation exchange chromatography and Ni^2+^ chelating column was successfully applied to purify RimJ into homogeneity. The single band in column 3 of Figure 2 corresponds to RimJ with a theoretical molecular weight of 22.6kDa.

### *In vitro* acetylation and mass spectrometry characterization

**Figure 3:**
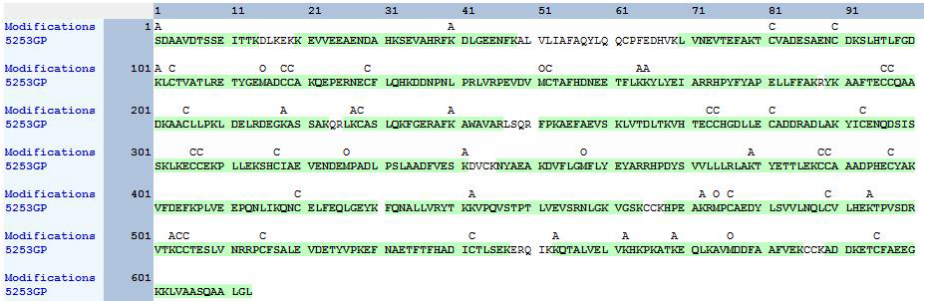
Sequence coverage and residue modification of Tα1-HSA detected by LC-MS/MS A: acetyle; C:carbamidomethyl; O: oxidation.

**Figure 4:**
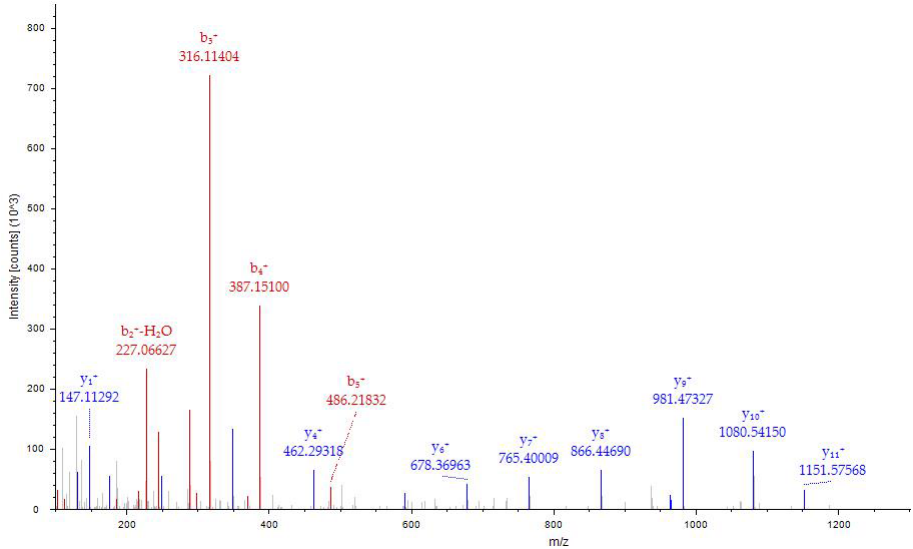
MS/MS spectrum of MH^+^1466.68767

*N^α^*-acetyl Tα1-HSA obtained after *in vitro* acetylation by RimJ was purified with Sephadex G50 and concentrated with centrifuge concentrators (Amicon Ultra-15, 50k MWCO). LC-MS/MS analysis covered 92.17% of the total sequence, including both the N- and C-termini of the fusion protein (Figure 3). The fusion protein had a native N terminus of Tα1 and a native C-terminus of HSA. A fragment with *m/z* of 1466.68767 was captured in the first stage of mass spectrometry and further analyzed as S(acetyl)DAAVDTSSEITTK in the second stage of mass spectrometry (Figure 4) since fragment b2^+^ is 42 larger than its un-acetylated counterpart. Apart from N-terminal acetylation on serine, acetylation on other 13 lysine residues of the fusion protein were also identified, which was speculated to be catalyzed by acetyltransferase in *P. pastoris*. The molecular mass of *N*^α^-acetyl Tα1-HSA was determined to be 69.5kDa.

*N^α^*-Acetylation is one of the most common protein modifications in eukaryotes, but rarely in prokaryotes [18]. However, previous studies have identified three NATs in *E.coli*, namely, RimL, RimJ and RimI, which are responsible for the acetylation of L12, S5 and S16 respectively [19, 20]. Fang *et al*. observed partial acetylation of fusion protein Tα1-L12 when it is recombinantly expressed in *E.coli*. They further disrupted the NATs in *E.coli* one by one and found that when RimJ was disrupted, the fusion protein was completely unacetylated. Based on this finding, we performed *in vitro* acetylation of Tα1-HSA by using purified RimJ. In the presence of AcCoA, a single acetyl group was added to the N-terminal serine residue of Tα1-HSA. Although it is possible to perform *in vivo* acetylation of Tα1-HSA if the fusion protein and RimJ are co-expressed in the host cell, complete *N*^α^-acetylation may not be achieved. The final product is very likely to be a mixture of *N^α^*-acetyl and *N^α^*-desacetyl Tα1-HSA, which are extremely difficult to be separated and purified into homogeneity.

### Kinetic study of *in vitro* acetylation

RimJ catalyzes the transfer of acetyl group from AcCoA to the α-NH_2_ of serine residue of Tα1-HSA or desacetyl Tα1. The resulting free CoA will react with DTDP to form a product with maximum absorption at 324 nm. The reaction rate was measured by the increase in OD_324_ of the reaction mixture. Theoretically, CoA and *N*^α^-acetyl Tα1-HSA or *N*^α^-acetyl Tα1 are formed in equal quantities over the whole time course of acetylation. According to Table 1, a smaller *K*_m_ value was observed for desacetyl Tα1, showing a higher affinity of RimJ toward Tα1. Higher *V*_max_ and *K*_cat_ for desacetyl Tα1 indicated that acetylation of desacetyl Tα1 could be more efficiently catalyzed by RimJ compared with Tα1-HSA. This difference was speculated to be caused by the larger molecular weight and spatial structure of the fusion protein that hindered the collision between enzyme and the substrate.

**Table 1:** Kinetic parameters of *in-vitro* acetylation of Tα1 and Tα1-HSA by RimJ

Substrate	*K*_m_(μM)	*V*_max_(M min^-1^)	*K*_cat_ (min^-1^)	*K*_cat_/*K*_m_(min^-1^ μM^-1^)
Tα1-HSA	0.58	10^-7^	0.13	0.22
desacetyl Tα1	0.26	1.49×10^-6^	0.22	0.85

### Bioactivity assay of *N^α^*-acetyl Tα1-HSA

Thymosin alpha 1 alone can't directly stimulate the proliferation of lymphocytes, but when used at low concentrations it can increase the proliferative response of lymphocytes to mitogens like phytohaemagglutinin (PHA) and concanavalin A (ConA). MTT proliferation assay was used to determine the growth-promoting effect of *N^α^*-acetyl Tα1-HSA, Tα1-HSA and Tα1(ZADAXIN^®^) on lymphocytes. HSA- Tα1 from our previous work was also assayed in parallel. As shown in Table 2, when used at different concentrations (0.75, 1.5, 3 and 6μM), all forms of Tα1 stimulated the proliferation of murine spleen lymphocytes in a dose dependent manner. In our previous work, we showed that fusion protein HSA-Tα1 and HSA-linker-Tα1, had comparable but slightly decreased growth promoting effect compared with Tα1(ZADAXIN^®^). The results are reproduced in Table 2, as indicated by the significant difference between the activity of Tα1(ZADAXIN^®^) and HSA-Tα1 at concentrations of 0.75, 3 and 6μM. When the position of Tα1 in the fusion protein was changed from C-terminus to N-terminus, Tα1activity was fully retained in the fusion protein. In the study by Daniela *et al*., injection of Tα1 or N_14_ fragment significantly increased the T cell precursor frequency in old mice, but no effect was induced by injection of the C_14_ fragment of Tα1, indicating that biological activity of the hormone was restricted to the first 14 amino acids [21]. This is one of the possible reasons for the fully retained activity of *N^α^*-acetyl Tα1-HSA. A fully exposed N-terminus of Tα1 in *N^α^*-acetyl Tα1-HSA is more favorable for the complete biological activity of the hormone.

**Table 2:** Growth-promoting effect of different forms of Tα1 on murine immunocyte (n=5)

	Concentration(μM)	Proliferation rate±SD(100%)
*N^α^*-acetyl Tα1-HSA	6.00	130.2±7.3
	3.00	80.1±4.2
	1.5	77.4±3.7
	0.75	51.9±1.9
Tα1-HSA	6.00	123.8±4.6
	3.00	87.0±3.7
	1.5	74.2±4.4
	0.75	50.3±2.8
HSA-Tα1	6.00	110.2±6.7^*^
	3.00	72.1±2.6^*^™
	1.5	65.4±3.4
	0.75	47.9±2.5^*^
Tα1(ZADAXIN^®^)	6.00	127.5±6.2
	3.00	84.6±4.7
	1.50	72.0±4.1
	0.75	54.6±2.3

Results are expressed as mean±SD (n=5), ^*^indicate statistically significant difference (*P* 0.05) as compared with activity of Tα1(ZADAXIN^®^) at the same concentration.

Tα1 is a *N*^α^-acetylated peptide that is mainly used as an immune-modulating agent to enhance the Th1 immune response. Chemically synthesized Tα1 is used worldwide for the treatment of some immunodeficiencies, malignancies, and infections. A biotechnological approach using the recombinant gene expression will be much more promising. In this study, we constructed fusion protein Tα1-HSA and *N^α^*-acetyltransferase RimJ, and purified them into homogeneity. *In vitro* acetylation of Tα1-HSA by RimJ was performed in the presence of AcCoA. LC-MS/MS identified the *N*^α^-acetylation on the N-terminus of Tα1, which is the natural form of the hormone and supposed to be involved in its *in vivo* stability. MTT proliferation assay indicated that the *in vitro* activity of Tα1 was fully retained in the fusion protein. *N*^α^-acetylation may further confer therapeutic advantages to *N^α^*-acetyl Tα1-HSA.

In eukaryotes, N-terminal acetylation of proteins is involved in the biological functions, stability and interactions with other proteins and/or peptide receptors. In the case of rat glycine N-methyltransferase, its N-terminal deacetylated form recombinantly produced in *E.coli* lacks the co-operative behavior of the native enzyme [22]. Similarly, the melanotropic action of α-melanocyte stimulating hormone (α-MSH) is increased by N-terminal acetylation [23]. However, early studies already showed that *N^α^*-acetylation did not affect the *in vitro* biological activities of Tα1 [13]. This was confirmed in our comparative study on the *in vitro* activity of Tα1-HSA and *N*^α^-acetyl Tα1-HSA. The N-terminal acetylation during or after the biosynthesis of eukaryotic proteins also serves to protect intracellular proteins from proteolysis. For example, enzymatic acetylation of the N-terminus of cytoplasmic actin converts the protein into a more stable form with insensitivity to aminopeptidase digestion [24]. Many researchers have proposed that *N^α^*-acetylation will affect the *in vivo* stability of Tα1. We also suspected that *N^α^*-acetylation will influence the *in vivo* stability even the *in vivo* activity of the newly obtained fusion protein Tα1-HSA, although the *in vitro* activity was not affected. In our previous work, fusion of partner HSA to Tα1 successfully increased the *in vivo* half-life of Tα1, which was advantageous for reducing dosing frequency and cost of treatment. Taking into consideration of the fully retained activity of Tα1 and potential stability benefits brought by *N^α^*-acetylation, *N^α^*-acetyl Tα1-HSA is likely to be a more promising therapeutic agent than Tα1(ZADAXIN^®^) and HSA-Tα1 or HSA-linker- Tα1. Comparative investigations on the *in vivo* stability and activity of *N*^α^-acetyl Tα1-HSA, Tα1-HSA and Tα1(ZADAXIN^®^) are underway to further explore the therapeutic potential of *N^α^*-acetyl Tα1-HSA and its advantages over Tα1-HSA and Tα1(ZADAXIN^®^).

## MATERIALS AND METHODS

### Microorganisms, vectors and materials

*P. pastoris* host strain GS115 and plasmid pPICZαA used for expression of Tα1-HSA were purchased from Invitrogen Co. (Shanghai, China). *E.coli* host strain BL21 and plasmid pET-28a(+) for expression of RimJ were purchased from EMD Biosciences (Novagen). *E. coli* DH5α and *E.coli* Top10 used for plasmid amplification were from TianGentech Co.(Beijing, China). Restriction endonucleases *Xho* I, *Not* I, *Nde* I, *Bam* HI and *Sac* I, DNA polymerases pfu, dNTP, and T4 DNA ligase were purchased from TaKaRa (TaKaRa Ltd., Dalian, China).

### Medium composition and culture conditions

Inocula of *E. coli* strains were cultured at 37°C and 200rpm in Luria-Bertani (LB) medium containing 0.5% (w/v) yeast extract, 1% (w/v) Tryptone and 1% (w/v) NaCl. To prepare the solid medium, 2% agar was added. When appropriate, zeocin (100 mg/ml) or kanamycin (25μg/ml) was added to the LB medium. For expression of the fusion protein by engineered strain GS115, BMGY medium was used to prepare seed culture and BMMY medium was used for flask fermentation.

### Construction of expression vector pPICZαA/Tα1-HSA

A codon-optimized full length Tα1-HSA gene was synthesized based on the protein sequence of Tα1 and HSA by GenScript Co., Ltd (Nanjing, China). Restriction sites *Xho* I and *Not* I were introduced at the 5’-and 3’-terminus respectively. In order to obtain a fusion protein with a native N-terminus, a Kex2 cleavage site was cloned directly downstream of the *Xho* I site to achieve precise incision of the signal peptide. After transformation into *E. coli* TOP10, positive clones were screened out by using 100μg/ml Zeocin. The target sequence was confirmed by double digestion and DNA sequencing.

### Transformation and screening of *P. pastoris* GS115 with high expression capacity of Tα1-HSA

Recombinant plasmid pPICZαA/Tα1-HSA amplified in *E. coli* TOP10 was linearized by *Sac* I, followed by transformation into competent *P.pastoris* GS115 prepared by treatment with lithium chloride. After plating onto YPD agar plate with 100 mg/ml zeocin and cultivation at 30°C for 48-72 h, positive clones were picked out and stocked in slant culture. The selected clones were first inoculated into 25 ml BMGY seed culture and incubated at 30°C until OD_600_ reached 10-15. The cell pellets were collected by centrifugation at 1500 rpm for 10 min and re-suspended in 50 ml BMMY medium. The fermentation was performed at 30°C in 500ml shaking flasks. The culture conditions were optimized by single factor experiments on days of induced expression (1-7 days) and methanol concentration (0.5-3.0%).

### Purification of Tα1-HSA

When the fermentation process was completed, the culture medium was harvested and centrifuged at 4°C and 8000 rpm for 20 min. The supernatant was concentrated by ultrafiltration using Millipore Cogent M1 Tangential Flow Filtration System (molecular weight cutoff, 30kDa) and then loaded onto a SephadeG-25 column(2.6cm×60cm) to remove pigment. A weak cation exchanger (Capto™ MMC, GE Health, 2cm×25cm) pre-equilibrated with sodium acetate -acetic acid buffer (25mM, pH 4.6) was then applied. The bound protein fractions were eluted using Na_2_HPO_4_-NaH_2_PO_4_ buffer (50mM, pH7.2) containing 1.0 M NH_4_Cl. Fractions containing the target protein were pooled and further loaded on a Blue-Sepharose™ 6 Fast Flow (GE Health, 2cm×25cm) column pre-equilibrated with 0.05M citric acid-0.1M Na_2_HPO_4_ (pH 7.0). The column was washed with the same buffer to baseline and the bound protein was eluted with 0.05M KH_2_PO_4_ containing 1.5M KCl (pH 7.0). The collection of target protein was stored at 4°C for further analysis. SDS-PAGE was carried out to determine the homogeneity of purification and the molecular mass of the recombinant fusion protein as previously described [25]. Coomassie brilliant blue R-250 was used for staining.

### Construction of expression vector pET-28a(+)/RimJ

The genomic DNA of *E.coli* DH5α was isolated. The complete open reading frame of RimJ gene was amplified using forward primer 5’-GGAATTCCATATGTTTGGCTATCGCAG-3’ and reverse primer 5’-CGCGGATCC TTAGCGGCCGGGCGTCCAGTC-3’ containing restriction sites *Nde* I and *Bam* HI, respectively. The PCR products were separated on 1% agarose gel electrophoresis and the resulting fragments were digested and inserted into pET-28a(+) vector, which produced RimJ fused to an N-terminal hexahistidine tag. After amplification in *E. coli* BL21, the target gene was confirmed by DNA sequencing.

### Expression and purification of RimJ

Transformed *E. coli* BL21 was allowed to grow in liquid LB medium overnight at 37°C and 200 rpm and then transferred into 50ml AIM medium in 500 ml shake flask. 12h after cultivation at 37°C and 200 rpm, cell pellet was obtained after centrifugation at 4000rpm for 20 min at 4°C and re-suspended in phosphate buffer (pH7.0). After washing for three times, the wet cell mass was sonicated for 20 min at 10°C. Supernatant was harvested by centrifugation at 12,000×g for 20 min at 4°C, and loaded onto a weak cation exchanger (Capto™ MMC, GE Health, 2cm×25cm) pre-equilibrated with PBS buffer (50 mM, pH 8.0). After elution with Na_2_CO_3_- NaHCO_3_ buffer (pH 10.0) containing 1.0M NH_4_Cl, fractions containing RimJ were collected and loaded onto a 10ml Ni^2+^ chelating Sephrose Fast Flow column. After washing with the binding buffer (50 mM sodium phosphate buffer, 500 mM NaCl, 50 mM imidazole, pH 7.5) to baseline, the bound protein was eluted by a washing buffer (50 mM sodium phosphate buffer, 500 mM NaCl, 500 mM imidazole, pH 7.5). Imidazole was then removed through dialysis against 50mM PBS (pH7.0).

### *In vitro* acetylation of Tα1-HSA by RimJ and mass spectrometry characterization

*In vitro* acetylation was carried out according to the previously reported method [15]. Reaction mixture contained 300μM 4,4'-DTDP(dithiodipyridine, AldrithiolTM-4), 50μM AcCoA (acetyl coenzyme A), 0.8μM RimJ and 50μM Tα1-HSA. Addition of 50mM PBS buffer (pH7.0) resulted in a final volume of 1 ml. Reaction mixtures were incubated for 12h at 25°C, followed by purification with Sephadex G50 (2.6cm×60cm) and concentration with centrifuge concentrators (Amicon Ultra-15, 50k MWCO). The resulting solution was loaded on a 12% gel. Protein band of expected molecular mass was incised, destained and subjected to in-gel tryptic digestion at 37°C overnight. The resulting peptides were extracted with 50% ACN/5% FA and then with 100% ACN. Peptides were dried and re-suspended in 2%ACN/0.1% FA. The peptides were separated by a reversed-phase analytical column (Acclaim PepMap RSLC, Thermo Scientific) and analyzed by Q Exactive™ Hybrid Quadrupole-Orbitra™ Plus Mass Spectrometer (ThermoFisher Scientific).

### Kinetic assays

Kinetic studies were conducted at 25°C in a total volume of 1ml. The reaction mixture contained 300μM 4,4'-DTDP, 50μM AcCoA, 0.8μM RimJ and different concentrations of Tα1-HSA (5-100μM). Reactions were started by adding RimJ into the reaction mixture. When the N-terminus of Tα1-HSA was acetylated, the acetyl-depleted form of AcCoA, CoA, began to react with DTDP and resulted in the reaction product with maximum absorption at 324 nm (ε_324_=19800 M^-1^cm^-1^). OD_324_ was measured every 3 minutes. Kinetic parameters *K*_m_, *V*_max_ and *K*_cat_ were calculated by the double reciprocal plot method. *In vitro* acetylation of desacetyl Tα1 was also investigated in parallel for comparison.

### Bioactivity assay of *N^α^*-acetyl Tα1-HSA *in vitro*

The growth-promoting effect of *N*^α^-acetyl Tα1-HSA on lymphocytes was determined by standard MTT (3-(4,5-dimethylthiazol-2-yl)-2,5-diphenyltetrazolium bromide) proliferation assay [26]. Spleen lymphocytes were prepared from 5 to 8 weeks old female BALB/c mice by pressing the animal spleen through a fine stainless mesh as previously described [27]. The cells were separated and suspended in RPMI 1640 medium containing 10% fetal bovine serum, 100 Uml^-1^ of penicillin and 100μg ml^-1^ of streptomycin and kept in an incubator with 5% CO_2_ at 37°C. Cells were seeded in a 96-well plates (4.0×10^5^/well) in the presence of 5μg/ml concanavalin A (ConA) for six hours. Then a serial dilutions of *N*^α^-acetyl Tα1-HSA were added to the wells. Cells stimulated with 5μg/ml concanavalin A alone was used as control. The activity of ZADAXIN, HSA-Tα1 and *N*^α^-desacetyl Tα1-HSA were also determined for comparison. After a total incubation of 72h, MTT solution was added to each well to a final concentration of 0.5 mg/ml and incubated for 4 hours. Supernatants in each well was discarded and 100 μL of DMSO was added to dissolve the formed crystal of formazan for about 10 min. Optical density of each well was measured at 570 nm using a Bio-Rad plate reader. Proliferation rate was calculated according to the following formula: proliferation rate = (OD_570__sample_- OD_570control_)/OD_570 control_. T-test was performed to evaluate statistical significance. All procedures involving animals have been approved by the Animal Ethics Committee in China pharmaceutical university.

## SUPPLEMENTARY MATERIALS FIGURES AND TABLES



## Abbreviations

AcCoA: acetyl coenzyme A
AIM: auto induction medium
BMGY: buffered glycerol-complex medium
BMMY: buffered methanol-complex medium
4,4'-DTDP: dithiodipyridine, AldrithiolTM-4
HAS: human serum albumin
LB: Luria-Bertani
phytohaemagglutinin: PHA
MTT: 3-(4,5-dimethylthiazol-2-yl)-2,5-diphenyltetrazolium bromide
Tα1: thymosin alpha 1
YPD: yeast extract-peptone-dextrose
