# Supplementary material for: Production of Nα-acetyl Tα1-HSA through in vitro acetylation by RimJ
Source: Oncotarget. 2017 Aug 14;8(56):95247–55. doi: 10.18632/oncotarget.20259 (PMC5707018; doi:10.18632/oncotarget.20259)
Supplement: Supplementary file 1 [file oncotarget-08-95247-s001.pdf]

Production of *N*$^{\alpha}$-acetyl Tα1-HSA through *in vitro* acetylation by RimJ

SUPPLEMENTARY MATERIALS

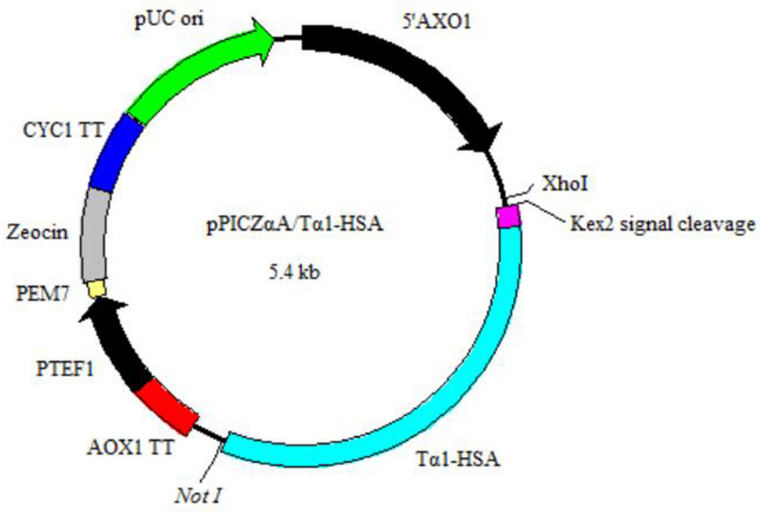

Supplementary Figure 1: Schematic map of recombinant expression vector pPICZαA/Tα1-HSA. The Tα1-HSA gene was inserted into pPICZαA using *Xho* I and *Not* I. Kex2 cleavage site was cloned directly downstream of the *Xho* I site.

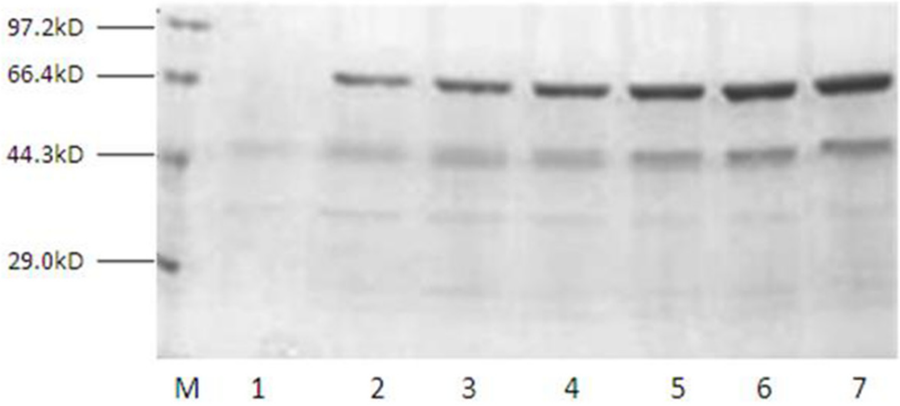

Supplementary Figure 2: Time course of Tα1-HSA expression after methanol induction. Lane M, marker; lane 1-7, expression after methanol induction for 1-7 days respectively.

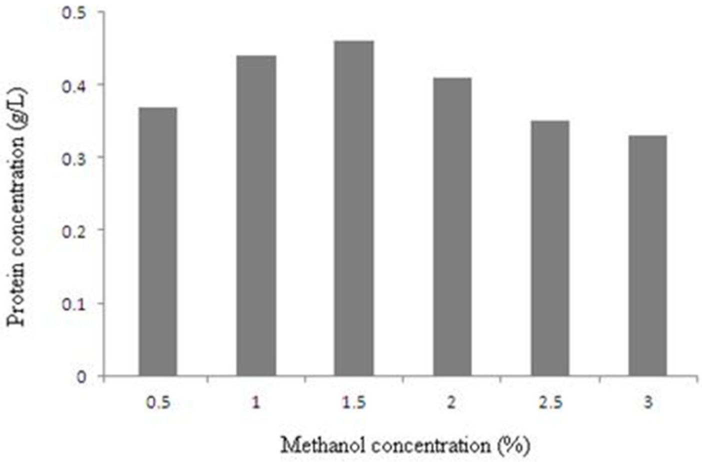

Supplementary Figure 3: Expression of Tα1-HSA after induction with different methanol concentrations for 6 days.
